# Within-Host Models of High and Low Pathogenic Influenza Virus Infections: The Role of Macrophages

**DOI:** 10.1371/journal.pone.0150568

**Published:** 2016-02-26

**Authors:** Kasia A. Pawelek, Daniel Dor, Cristian Salmeron, Andreas Handel

**Affiliations:** 1 Department of Mathematics and Computational Science, University of South Carolina Beaufort, Bluffton, South Carolina, United States of America; 2 Department of Natural Sciences, University of South Carolina Beaufort, Bluffton, South Carolina, United States of America; 3 Department of Epidemiology and Biostatistics, College of Public Health, University of Georgia, Athens, Georgia, United States of America; Indiana University, UNITED STATES

## Abstract

The World Health Organization identifies influenza as a major public health problem. While the strains commonly circulating in humans usually do not cause severe pathogenicity in healthy adults, some strains that have infected humans, such as H5N1, can cause high morbidity and mortality. Based on the severity of the disease, influenza viruses are sometimes categorized as either being highly pathogenic (HP) or having low pathogenicity (LP). The reasons why some strains are LP and others HP are not fully understood. While there are likely multiple mechanisms of interaction between the virus and the immune response that determine LP versus HP outcomes, we focus here on one component, namely macrophages (MP). There is some evidence that MP may both help fight the infection and become productively infected with HP influenza viruses. We developed mathematical models for influenza infections which explicitly included the dynamics and action of MP. We fit these models to viral load and macrophage count data from experimental infections of mice with LP and HP strains. Our results suggest that MP may not only help fight an influenza infection but may contribute to virus production in infections with HP viruses. We also explored the impact of combination therapies with antivirals and anti-inflammatory drugs on HP infections. Our study suggests a possible mechanism of MP in determining HP versus LP outcomes, and how different interventions might affect infection dynamics.

## Introduction

The World Health Organization (WHO) identifies influenza as a major public health problem [1]. Every year people get infected with seasonal, zoonotic, or pandemic strains of influenza. Influenza strains can be categorized as having either low pathogenicity (LP) or high pathogenicity (HP), which refers to the ability of the virus to induce disease in a specific host. Infections with HP avian influenza have led to severe complications in children and young adults [2, 3]. A recent outbreak of an H7N9 avian influenza strain occurred in China during the spring of 2013 and was reported to have caused 135 human cases and 44 deaths [4]. The most catastrophic influenza related pandemic, caused by an H1N1 strain with an increased pathogenicity (several times the mortality of typical seasonal strains), occurred in 1918 and caused approximately 500 million infection cases and an estimated 50–100 million deaths [5]. Infections with low pathogenicity influenza viruses are less likely to cause severe illness or lead to the death of the infected individual. A better understanding of the mechanisms that may lead to severe infections caused by the HP viruses would be very valuable in our continued efforts to combat influenza.

Because of the limitations associated with data collection during human HP influenza infections, animal experiments and models are essential in obtaining a better understanding of viral load regulation within a host. Numerous mammalian models have been examined to investigate avian influenza development; they have provided us with crucial information about the disease [6–10]. Complementing these experimental studies, mathematical models can provide insight into understanding infection dynamics and the role of immune response in controlling the disease or leading to complications in certain cases of the disease.

A number of mathematical models have been developed to study the dynamics of uncomplicated influenza virus infection and immune responses [11–21]. A recent modeling study showed that a model with two types of susceptible cells could explain elevated viral titer in HP infections and provide a more realistic fit to HP viral load data than a model with one cell type [22]. However, it was not specified what type of cells were represented by the second cell population in the model.

Here, we further explore this idea of a secondary population of cells that can be productively infected. We specifically focus on the role of macrophages. Macrophages constitute an important component of the innate immune response and have been shown to have an important role during influenza infections [23–27]. However, their role in HP influenza infections is still uncertain.

In [28] it was shown that in fatal infections with HP H1N1 and H5N1 influenza viruses high numbers of macrophages and neutrophils are expressed in the lungs. This study also performed experiments *in vitro*, showing that primary macrophages and dendritic cells are susceptible to HP virus infection [28]. Other studies have also shown that macrophages can be productively infected with influenza viruses [29, 30]. In [29], it was shown that the H5N1 virus can productively replicate in alveolar macrophages. Furthermore, primary human macrophages infected with avian H5N1 resulted in more efficient productive replication than infection with human influenza viruses [30]. The viral replication competence of macrophages and their contribution to overall functions in the pathogenesis of the infection with HP viruses are not fully understood. In our modeling study we explored protective and pathogenic functions of macrophages and highlight their possible role as cells that contribute both to immune response function and virus production. After showing that our model can capture the dynamics of HP infections, we use our model to explore the impact of drugs on HP infection dynamics.

## Materials and Methods

### 1. Mathematical model

We developed a mathematical model based on differential equations to study the within-host dynamics of influenza infection. The model has seven variables: uninfected epithelial cells susceptible to infection (*T*), productively infected epithelial cells (*I*), free virus (*V*), uninfected resting macrophages circulating within the host (*M*_*R*_), activated macrophages at the site of the infection (*M*_*A*_), productively infected macrophages (*M*_*I*_), and the antibody/B-cell component of the immune response (*A*).

**Fig 1:**
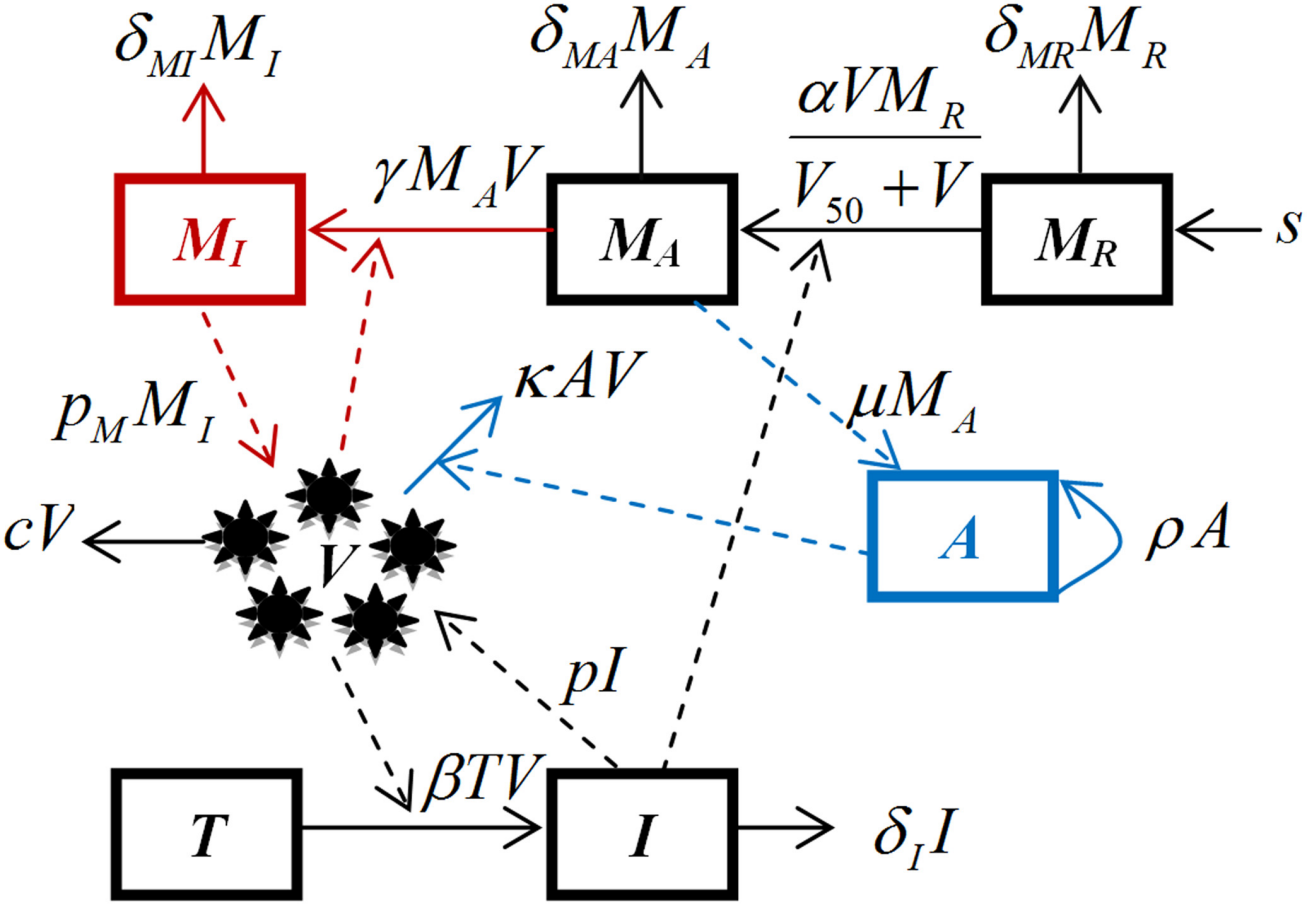
Schematic representation of the full model. A detailed description of the model, the set of differential equations, and meaning and values for variables, and parameters are given in the Materials and Methods section and Tables 1 and 2.

A schematic diagram of the model is shown in Fig 1. Variables and parameters are summarized in Tables 1 and 2, respectively. The mathematical formulation of the model is given by the following set of ordinary differential equations:

$$\begin{array}{l} \frac{\text{d}T}{\text{d}t}=-\beta TV\\ \frac{dI}{dt}=\beta TV-\delta_{I}I\\ \frac{dV}{dt}=(1-\varepsilon_{1})(pI+p_{M}M_{I})-cV-\kappa AV-\beta TV\\ \frac{dM_{R}}{dt}=s-(1-\varepsilon_{2})\alpha VM_{R}/(V_{50}+V)-\delta_{MR}M_{R}\\ \frac{dM_{A}}{dt}=(1-\varepsilon_{2})\alpha VM_{R}/(V_{50}+V)-\gamma M_{A}V-\delta_{MA}M_{A}\\ \frac{dM_{I}}{dt}=\gamma M_{A}V-\delta_{MI}M_{I}\\ \frac{dA}{dt}=\mu M_{A}+\rho A \end{array}$$

**Table 1:** Definitions of variables used in the model and their initial values.

Variable	Definition	Initial Condition
*T*	Uninfected epithelial cells susceptible to infection	7×10^9^, [12]
*I*	Infected epithelial cells	0
*M*_*R*_	Uninfected macrophages circulating the host	*s/δ*_*MR*_, [31]
*M*_*A*_	Activated macrophages at the infection site	0
*M*_*I*_	Productively infected macrophages	0
*V*	Virus	1×10^2^, as reported in [28]
*A*	Immune response due to antibodies	0

**Table 2:** Parameter definitions, units, values, and references.

Symbol	Definition	Unit	Value and Reference
*δ*_*MR*_	Death rate of uninfected macrophages, *M*_*R*_	day^-1^	1/25, mean based on [31]
*δ*_*MA*_	Death rate of activated macrophages at the infection site, *M*_*A*_	day^-1^	see text
*δ*_*MI*_	Death rate of infected macrophages, *M*_*I*_	day^-1^	see text
*s*	Constant generation rate of uninfected macrophages circulating within a host (*M*_*R*_)	cells day^-1^	fitted
*δ*_*I*_	Death rate of infected epithelial cells	day^-1^	2, [10, 20]
*β*	Infection rate of epithelial cells	(PFU)^-1^ ml day^-1^	fitted
*α*	Maximum recruitment rate of macrophages to the site of infection	day^-1^	fitted
*V*_*50*_	Virus load at which activation reaches half its maximum	(PFU)^-1^ ml	fitted
*γ*	Infection rate of macrophages	(PFU)^-1^ ml day^-1^	fitted
*p*	Virus production rate from epithelial cells	PFU (ml)^-1^ day^-1^ cell^-1^	fitted
*p*_*M*_	Virus production rate from macrophages	PFU (ml)^-1^ day^-1^ cell^-1^	fitted
*μ*	Activation of immune response	day^-1^	fitted
*ρ*	Rate of expansion of B-cells	day^-1^	1, [12]
*κ*	Clearance rate of free virus due to immune system	day^-1^ cell^-1^	fitted
*c*	Virus clearance rate due to mechanisms other than antibodies	day^-1^	3, [11, 13]
*ε*_1_	Antiviral treatment efficacy	-	0–1
*ε*_2_	Anti-inflammatory treatment efficacy	-	0–1

Infection of susceptible epithelial cells is described by the term *βVT*, which represents the rate of encounter with virus and subsequent infection. Infected cells die at a rate *δ*_*I*_. Virus particles are produced by infected epithelial cells at rate *p*. Additional production of virus by infected macrophages occurs at rate *p*_*M*._. Virus is cleared by the B-cell/antibody immune response at rate *κAV*. Additional, non-antibody specific virus clearance occurs at the rate *c*.

Non-activated macrophages (*M*_*R*_) are assumed to be generated at the constant rate *s* and to die at rate *δ*_*MR*_ [31]. The term *αVM*_*R*_/(*V*_*50*_*+V*) represents the rate at which macrophages are activated. This activation is assumed to be proportional to the viral load, with a maximum activation at the rate *α*, the parameter *V*_*50*_ represents the viral load at which activation reaches half its maximum. The activated macrophages at the site of infection (*M*_*A*_) help activate the adaptive immune response [24, 26]. Mechanistically, this activation is likely indirect, with MP producing pro-inflammatory cytokines and chemokines, which in turn help activate other cells of the innate response, e.g. dendritic cells, and the adaptive response. To keep our model simple, we describe this indirect, multi-step interaction from MP to adaptive response by a direct activation rate, which we model to occur proportional to the number of activated MP at rate *μM*_*A*_.

Activated macrophages die at rate *δ*_*MA*_ and, in the case of infection with HP viruses, activated macrophages at the site of the infection can be infected [28–30], which we model to occur at rate *γM*_*A*_*V*. Infected macrophages are assumed to lose their ability to help activate the adaptive immune response and instead start producing virus [28–30] at rate *p*_*M*_. Infected macrophages die at rate *δ*_*MI*_. B-cells that have been activated proportional to the number of macrophages grow exponentially through division at rate *ρ*. Since we are only interested in the acute infection dynamics, we do not model contraction of the adaptive immune response after clearance of the infection.

B-cells/antibodies clear virus particles [32], which in our model occurs at the rate *κAV*. Note that we simply assume that antibodies are proportional to B-cells and therefore do not use two separate equations for B-cells and antibodies but instead combine them in one equation.

Our hypothesis is that LP and HP infections differ, in part, due to HP viruses’ capability to productively infect macrophages. To show this in our model, we run simulations for the LP scenarios with parameters γ and *p*_*M*_ set to zero, i.e. no infection of and subsequent virus production by macrophages occurs. In contrast, for the HP scenarios, these parameters are allowed to be non-zero.

We also investigate the effect of two different potential treatments that might be given to combat HP influenza infections. In particular, we incorporated the effect of neuraminidase inhibitors (oseltamivir and zanamivir), which are widely used against influenza infection [33]. Similarly to previous modeling studies [13, 34] we introduce the antiviral effect of the neuraminidase by lowering the viral production by a factor of (1 − *ε*_1_), where *ε*_1_ is the drug efficacy. We further model the effect of an anti-inflammatory drug which inhibits the activation and recruitment of macrophages to the site of infection, with efficacy *ε*_2_.

### 2. Experimental data

We compared our model to data from experimental influenza infection studies of BALB/c mice infected with LP viruses: TX/91 (H1N1) and SP/83 (H5N1) and HP viruses: 1918 (H1N1) and Thai/16 (H5N1) [28]. Specifically, viral load and macrophage data were extracted from Figs 1 and 2 in [28] using Engauge Digitizer (digitizer.sourceforge.net). For further details about the data see the original study.

It is worth noting that the markers used in [28] to identify macrophages might not have captured all subtypes of macrophages and further might include subpopulations of cells that are not classically defined as macrophages. The difficulty of cleanly defining and counting macrophages based on specific markers is a limitation of the available data. For our study purposes we assume that the measured cells represent the bulk of the activated macrophages. However, this caveat with regard to the experimental data needs to be kept in mind.

### 3. Parameter values and data fitting

To avoid over fitting of the models, some of the parameters were fixed, with values taken from the existing literature. The lifespan of infected epithelial cells, 1/*δ*_*I*_, was fixed at 0.5 days in agreement with previous modeling studies [10, 20, 35]. The initial population of epithelial cells in the mice lungs was fixed at 7×10^9^ cells based on a value provided in [12]. We set the initial population of infected epithelial cells and infected macrophages to 0. Following [31], we set the initial number of uninfected, resting macrophages to *M*_*R*_*(0) = s/δ*_*MR*_. Death rates of macrophages in the different states, (*δ*_*MR*_, *δ*_*MA*_, and *δ*_*MI*_), are assumed to be equal due to the lack of data and their value is taken to be 1/25 day^-1^ based on [31]. Virus clearance rate due to mechanisms other than antibodies, *c*, was set to 3 day^-1^, following [11, 13]. The rate of expansion of B-cells, *ρ*, was set to 1 day^-1^, based on [12].

The remaining parameters were estimated by fitting the model to the data described above. Specifically, for the H1N1, as well as, the H5N1 viruses studied in [28], we fit viral load and macrophage data for the LP and HP strains simultaneously to the same model, with the difference being that the parameters describing macrophage infection and virus production (*γ* and *p*_*M*_, respectively) are zero for LP and non-zero for HP. Additionally, LP and HP strains were allowed to vary in their rate of activation of macrophages, *α*. All other parameter values are shared between the LP and HP scenarios. This allows us to test our hypothesis that differential activation and productive infection of macrophages can explain the observed differences between LP and HP infections.

To allow simultaneous fitting of two different experimental quantities, namely viral load and macrophage numbers, we follow [21] and fit the model by minimizing the weighted sum of square differences, with weights used to standardize viral load and macrophage contributions and allow for joint summation. The objective function that we minimize is given by the following equation:

$$SSR=\sum_{i=1}^{n_{V}}{\left(\frac{{\text{log}}_{10}V_{i}^{m}-{\text{log}}_{10}V_{i}}{{\text{log}}_{10}V_{\text{max}}}\right)}^{2}+\sum_{i=1}^{n_{M}}{\left(\frac{{\text{log}}_{10}{\left(M_{A}^{m}+M_{I}^{m}\right)}_{i}-{\text{log}}_{10}M_{i}}{{\text{log}}_{10}M_{\text{max}}}\right)}^{2}\;$$

Where viral load data (for both LP and HP strains) is given by *V*_*i*_ and the corresponding value predicted by the model is *V*_*i*_^*m*^. Macrophage data is given by *M*_*i*_ and the analogous model prediction for the sum of the activated and infected macrophages at the site of the infection is represented by (*M*_*A*_^*m*^*+M*_*I*_^*m*^*)*_*i*_. The maximum data values of the viral load and macrophages are denoted by *V*_*max*_ and *M*_*max*_, respectively.

To deal with viral load data that is at or below the limit of detection (left-censored), we keep the squared difference if the model predicts a value above the limit of detection, but set any difference to zero for a model prediction that is below the limit of detection (10^0.5^ PFU/ml). Lastly, since the reported data did not track the infection all the way to its conclusion, we augmented the data by adding a value for the virus load at the limit of detection 15 days post infection. This was required to ensure fits that agree with the known biology of the infection dynamics.

We used R Version 3.2. [36], as well as, the packages nloptr [37] and deSolve [38] to fit the model to the data. Packages dplyr [39] and ggplot2 [40] were also used. The data and R scripts to reproduce all results are provided as supplementary material.

## Results

### Productive infection of macrophages can explain HP infection dynamics

**Fig 2:**
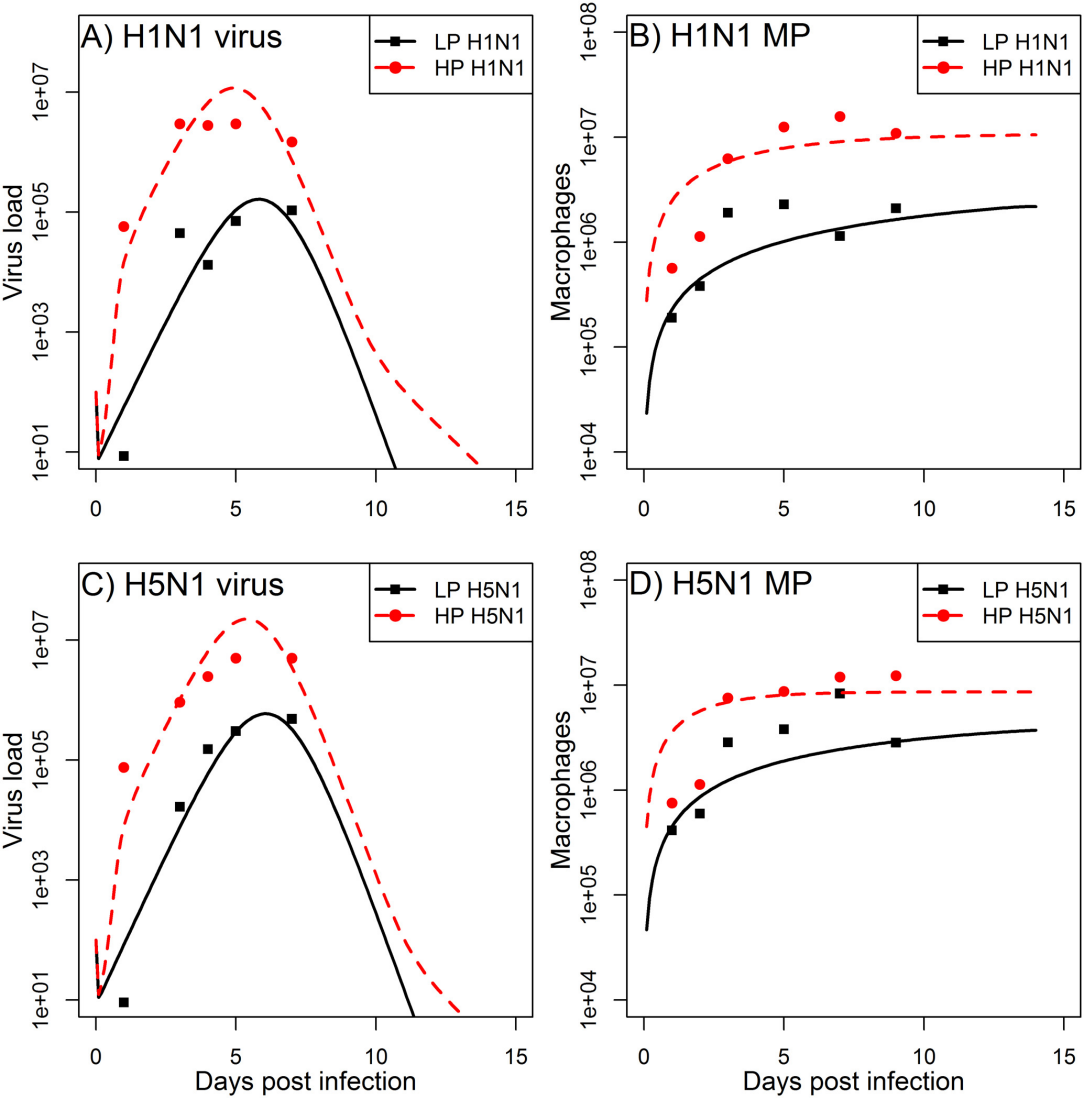
Best fits of the model to the experimental data for viral load and macrophages reported in [28]. Macrophages as predicted by the model are *M*_*A*_*+M*_*I*_. Parameter values for the best fit estimates are listed in Tables 2 and 3.

Dobrovolny et. al. showed that the cell tropism may explain the observed disease severity of influenza infections caused by HP viruses [22]. Their model included two cell populations, which have different susceptibility to the infection and virus production rates, and was fitted to viral load data [22]. While [22] envisioned these two cell types to be different types of epithelial cells, here we consider the possibility that the second cell type are macrophages, which can both become productively infected and also play a role in combating the infection. We fit the model to both viral load and macrophage data and explore the hypothesis that macrophages may have both protective and pathogenic role in an HP influenza infection. To test our hypothesis we simultaneously fitted our model to LP and HP infection data, with the only difference being that the parameters describing macrophage activation, infection, and virus production (*α*, *γ*, and *p*_*M*_, respectively) differ between LP and HP, with the latter two being zero for LP. Such a model provides a reasonable fit to the data (Fig 2). This suggests that our hypothesis that the productive infection of macrophages can explain the observed differences between LP and HP infections is plausible.

**Fig 3:**
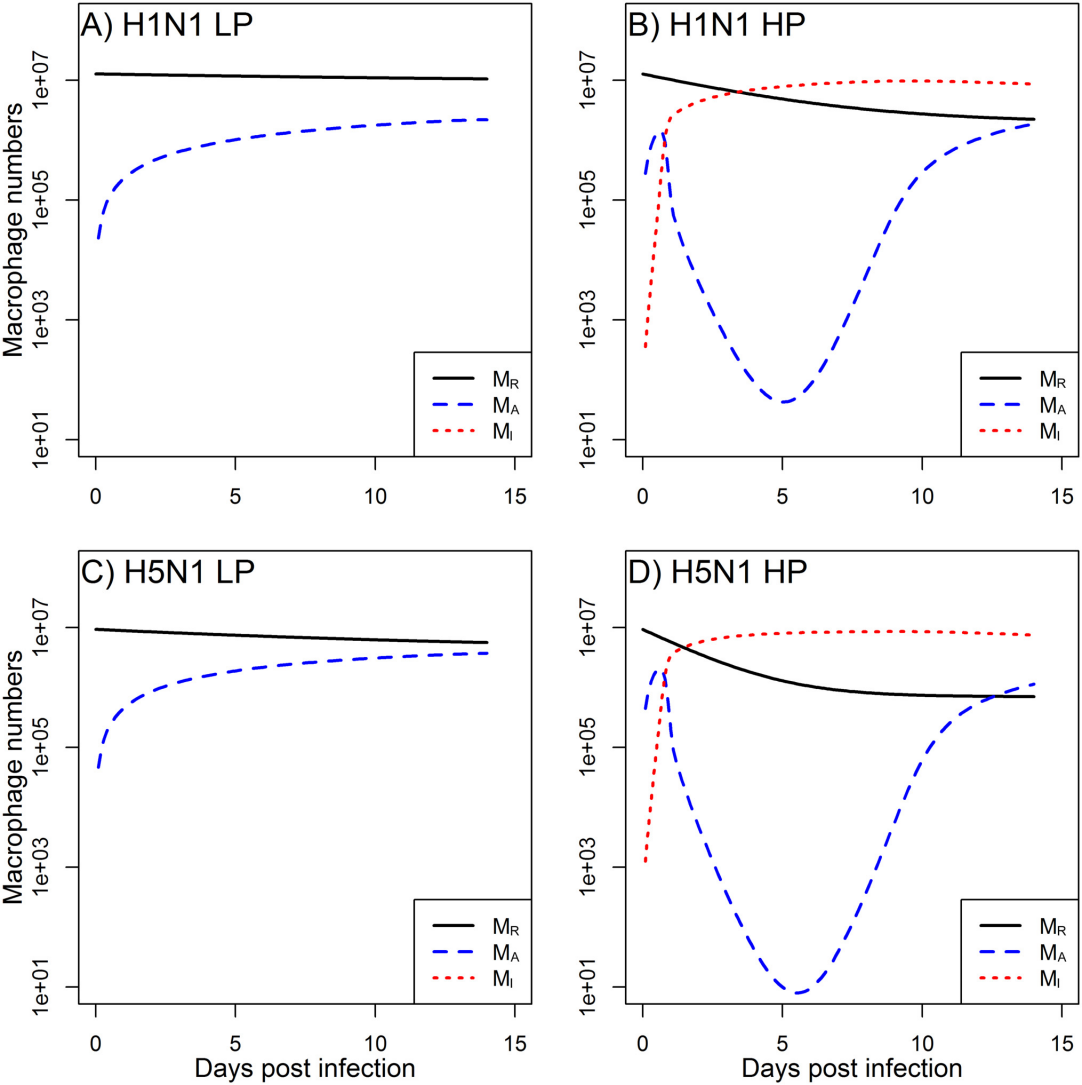
Model predictions for non-activated macrophages (*M*_*R*_), activated macrophages (*M*_*A*_), and productively infected macrophages (*M*_*I*_). Figure shows the LP and HP H1N1 and H5N1 scenarios corresponding to the viral load and total macrophage model results shown in Fig 2. Parameter values are listed in Tables 2 and 3.

Furthermore, our modeling predictions suggest that macrophages become activated quickly in both LP and HP infections (Fig 3). In particular, HP infections lead to greater activation of MP. However, the majority of these activated MP become infected (Fig 3), and therefore are not able to properly participate in the immune response, subsequently leading to the increased viral load seen in the data (Fig 2).

**Fig 4:**
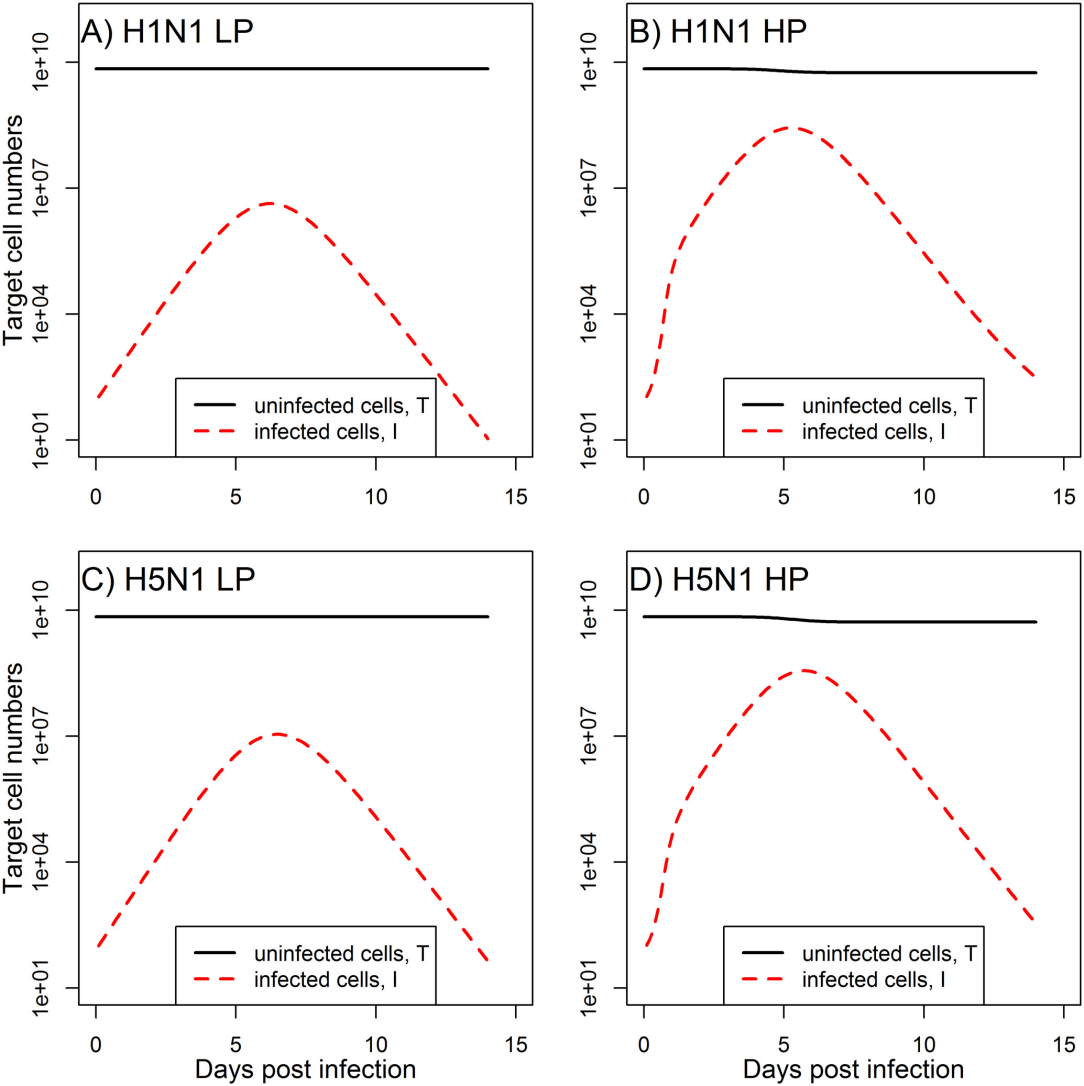
Model predictions for the susceptible epithelial cells (*T*) and infected epithelial cells (*I*). The figure shows the LP and HP H1N1 and H5N1 scenarios corresponding to the viral load and macrophage model results shown in Figs 2 and 3. Parameter values are listed in Tables 2 and 3.

As can be seen in Fig 4, our model predicts that the decline in target cells is not considerable. This agrees with experimental evidence of influenza infections, which have shown that—at least in mice—destruction of approximately more than 10% of alveolar type I cells leads to severe pathology and host death [41]. Models which do not include immune response and where removal of target cells is the only mechanism by which an infection can end predict depletion of target cells beyond biologically reasonable levels, a feature that has been discussed previously [20, 42].

### Impact of HP model parameters on viral load and macrophage response

**Fig 5:**
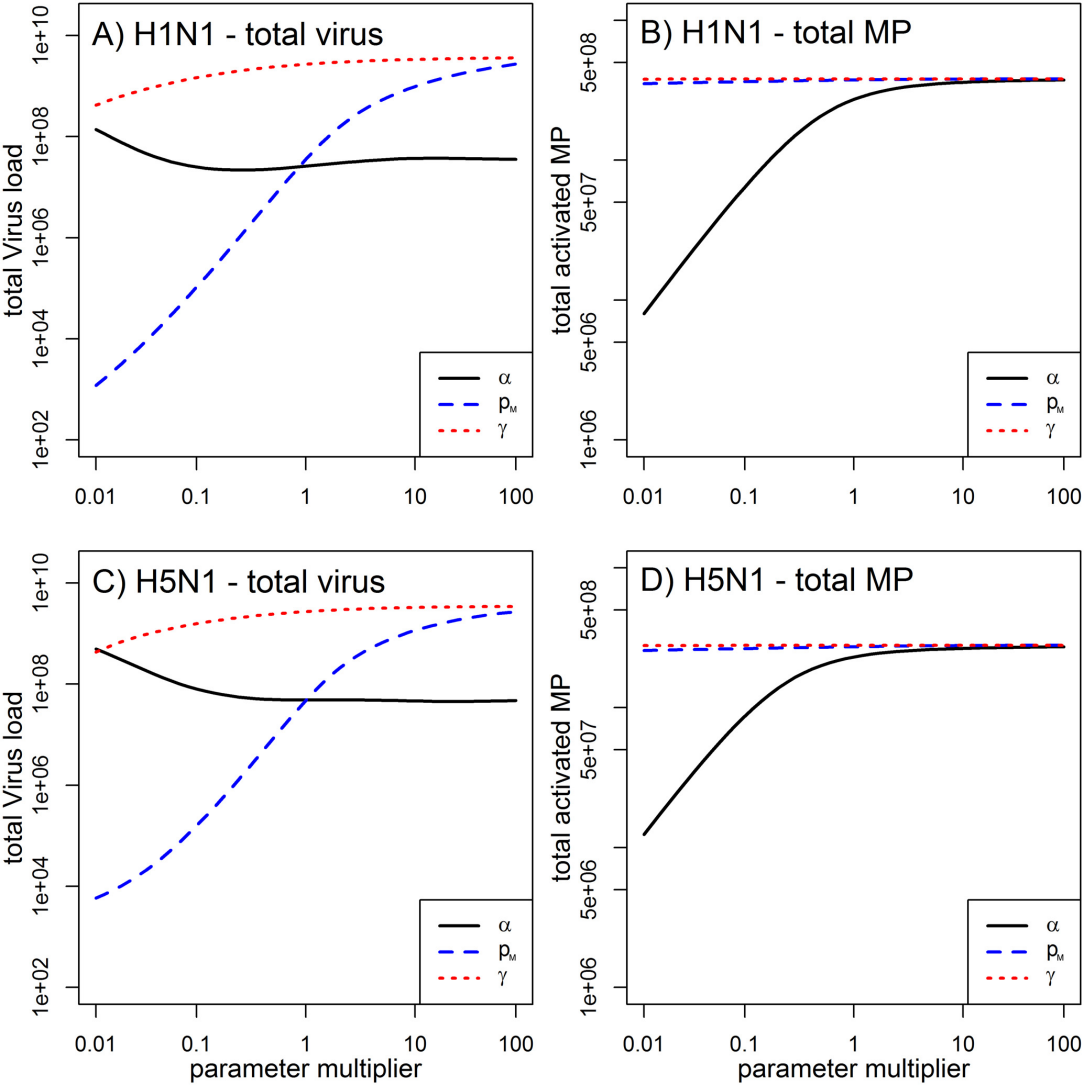
Impact of HP associated parameter values on total viral load and total activated macrophages. We individually varied each of the parameters *α*, *p*_*M*_, and *γ* in a range of 0.01–100 times its original value for HP infections shown in Table 3. All other parameters were kept at the values reported for the HP scenario in Tables 2 and 3.

We performed a sensitivity analysis to look more closely at the 3 parameters associated with HP infections in our model, namely *α*, γ, and *p*_*M*_. For both H1N1 and H5N1 strains, we simulated the model and varied each of these 3 parameters individually in a range of 0.01–100 times the original parameter obtained from the best fit (Table 3). All other parameters were kept at their best fit estimates for the HP strain. For each run, we computed two measures to summarize the infection. As one measure, we followed [43, 44] and used total viral load (area under curve). As a second measure, we computed the total number of activated macrophages during the infection. The latter quantity can be thought of as representing a proxy for the amount of inflammation/immune pathology present during the infection. As Fig 5 shows, increased virus production by MP (increased *p*_*M*_) leads to a higher viral load (Fig 5A and 5C), but as expected, does not impact the number of activated MP (Fig 5B and 5D). The rate at which MP become infected (*γ*) has a similar effect; however has less effect on the viral load increase than the result of the increase of the parameter *p*_*M*_ (Fig 5A and 5C). The only parameter that affects the number of MP is the rate of MP activation (*α*) (Fig 5B and 5D). This rate has little impact on total viral load, with initial increases in MP activation leading to reduced viral load, up to some level after which activation rate has limited further impact.

**Table 3:** Best fit parameter values to the viral load and macrophage data. The best fits are displayed in Fig 2.

Virus	*β*	*p*	*κ*	*μ*	s	*V*_*50*_	*α* for LP	*α* for HP	*p*_*M*_	*γ*
H1N1	8.4 × 10^−9^	4.6	2.6 × 10^2^	2.5 × 10^−9^	508725.2	3.9 × 10^−4^	1.8 × 10^−2^	2.2 × 10^−1^	2.0 × 10^−1^	2.2 × 10^−3^
H5N1	6.0 × 10^−9^	4.9	2.2 × 10^−9^	9.7 × 10^1^	372997.0	3.4 × 10^−4^	5.0 × 10^−2^	4.9 × 10^−1^	5.4 × 10^−2^	3.2 × 10^−3^

### Antiviral and anti-inflammatory therapies in infections with HP viruses

**Fig 6:**
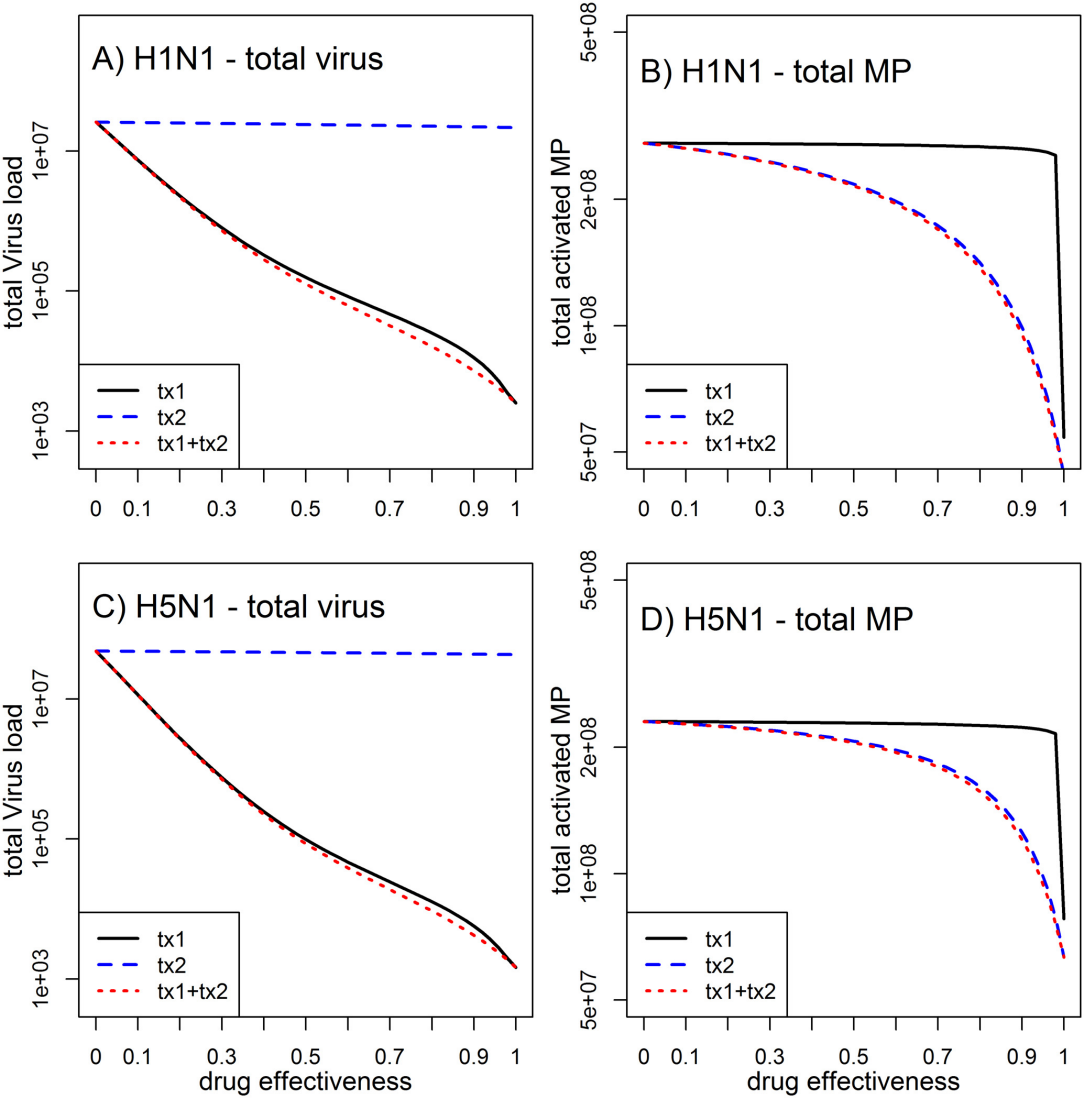
Impact of different treatment strategies on total viral load and total activated macrophages. Antiviral treatment (tx1), anti-inflammatory treatment (tx2) or both (tx1+tx2) were systematically varied between no effectiveness (*ε*_1_ and/or *ε*_2_ at 0) and 100% effectiveness (*ε*_1_ and/or *ε*_2_ at 1). The remaining parameters are set to the HP scenarios listed in Tables 2 and 3.

We added the effect of two drugs to our model to study how either antiviral or anti-inflammation treatment, or the combination of the two, would affect the infection dynamics. Fig 6A and 6C shows that treatment with antivirals, such as neuraminidase inhibitors, leads to a reduction of viral load, which is in line with both experimental data and previous modeling studies [13, 34]. However, for most treatment efficacies, the antiviral drug has little impact on activation of macrophages (Fig 6B and 6D), which we consider here a proxy for immune mediated inflammation and morbidity. This is somewhat reminiscent of observations from treatment of regular (non HP) influenza with neuraminidase antivirals in humans, where there is a clear impact on viral load but relatively modest impact on symptoms [36, 45–47]. Our model predicts that only at efficacies >90% the suppression of viral load leads to a subsequent reduction in macrophage activation. In contrast, treatment targeting macrophage activation has essentially no impact on viral load (Fig 6A and 6C), but reduces the total number of activated macrophages even at intermediate values of treatment effectiveness (Fig 6B and 6D). The combination of both drug treatments works additively, with the antiviral reducing viral load and the anti-macrophage activation reducing total number of activated macrophage. Our model suggest that this combined treatment approach, which has been suggested previously [48, 49], seems to be the most promising in overall targeting HP influenza infections.

We considered here a very optimistic scenario where treatment occurs 1 day post infection. Clinically, this is hard to achieve. Obviously, a later start of treatment reduces the impact of both interventions. Starting treatment 2 days post infection or later leads to little impact on virus load and macrophage numbers, even at high efficacies (simulations not shown). This agrees with the general understanding that rapid intervention post infection is crucial for maximum impact.

## Discussion

Understanding why some influenza strains cause relatively little disease and pathology, while others often lead to severe outcomes, is important if we want to further improve our ability to control influenza. Here, we explored the hypothesis that macrophages, that can have both a protective effect and, through becoming infected, contribute to pathology, might be one mechanism that distinguishes LP from HP influenza infections. Macrophages are at the forefront in the defense against foreign invasion by micro-organisms. During an influenza infection, activated macrophages in the lungs stimulate cytokines, TNFα and TNFβ, which recruit additional macrophages and other immune system cells that play a role in the recognition of foreign antigens and support proper activation of adaptive response. When macrophages recognize invading particles, they confront the invaders and release chemical signals which activate the immune response. Macrophages attempt to clear the virus by phagocytosis [50–52]. This may be the mode by which macrophages could become infected and contribute to the total viral load during HP influenza infections.

By developing a mathematical model and fitting it to virus and macrophage data for LP and HP infections, we were able to test whether a difference in parameters associated with macrophage activation, infection, and virus production can explain observed differences between LP and HP virus infections. Our modeling results suggest that the productive infection of macrophages with HP influenza viruses is a plausible explanation for the different infection outcomes observed in mice infected with LP and HP virus strains. Our finding for MP contributing both to virus clearance and pathology mirrors a recent finding that dendritic cells can be a double-edged sword in influenza infections [53].

Using our model parameterized for HP infections, we investigated the impact of antiviral and anti-inflammatory drugs. We find that while antiviral drugs can reduce virus load, the impact on pathology (which we quantified with macrophage numbers as proxy) is minimal. Some experimental studies have reported similar minimal benefits of antiviral therapies during HP influenza infection [54, 55].

Alternatives to antiviral drugs targeting the virus are anti-inflammatory approches. For instance, anti-TNF agents currently on the market may prove efficient in controlling the immune response [56] and thus reducing pathology during influenza infections. Our model predicted an effect of anti-inflammatory drugs in substantially lowering the number of macrophages at the site of the infection, however there was minimal impact on viral load. If both antiviral and anti-inflammatory drugs were combined, our model predicted additive effects leading to reduction in both viral load and pathology.

The usual caveats to our study apply. The fact that our model can explain the experimentally observed differences between LP and HP infections should only be taken as supportive, not confirmatory, of the idea that macrophages play such a double-edged role in virus clearance and pathology. Many other models may also adequately describe the observed data. Further, the data are from infections in mice. While influenza infections in mice capture some of the characteristics of human influenza infections, there are important differences and it is unclear what role MP play for human influenza infections.
