# Correction: Within-Host Models of High and Low Pathogenic Influenza Virus Infections: The Role of Macrophages

**DOI:** 10.1371/journal.pone.0215700

**Published:** 2019-04-16

**Authors:** Kasia A. Pawelek, Daniel Dor, Cristian Salmeron, Andreas Handel

In this study [1], the authors ran their model in infectious virion units, and assumed that one infectious virion corresponds to 1 PFU. This assumption was not stated clearly. It applies for setting the initial condition to 100 virions (corresponding to 100 PFU reported in the experimental data), when fitting the model to the data and plotting model and data virus load.

Following this point, Table 2 in [1] mis-reports units for some parameters. Any mention of PFU or mL (or the inverse of those) should not be present in the unit column of this table.

Finally, the raw data underlying this study and the R code file(s) needed to replicate the results are appended to this Correction as S1 File and S2 File, respectively.

## Supporting information

S1 File Raw data. (ZIP)

S2 File R code. (ZIP)
